# Application of a Dot Blot Hybridization Platform to Assess *Streptococcus uberis* Population Structure in Dairy Herds

**DOI:** 10.3389/fmicb.2017.00054

**Published:** 2017-01-24

**Authors:** Pedro Albuquerque, Niza Ribeiro, Alexandre Almeida, Irena Panschin, Afonso Porfirio, Marta Vales, Francisca Diniz, Helena Madeira, Fernando Tavares

**Affiliations:** ^1^CIBIO, Centro de Investigação em Biodiversidade e Recursos Genéticos, InBIO, Laboratório Associado, Universidade do PortoVairão, Portugal; ^2^Instituto de Ciências Biomédicas de Abel Salazar, Universidade do PortoPorto, Portugal; ^3^Estudos de Populações, Instituto de Saúde Pública da Universidade do PortoPorto, Portugal; ^4^Departamento de Biologia, Faculdade de Ciências, Universidade do PortoPorto, Portugal

**Keywords:** *Streptococcus uberis*, bovine mastitis, dot blot, multilocus sequence analysis, population structure

## Abstract

*Streptococcus uberis* is considered one of the most important pathogens associated with bovine mastitis. While traditionally acknowledged as an environmental pathogen, *S. uberis* has been shown to adopt a contagious epidemiological pattern in several dairy herds. Since different control strategies are employed depending on the mode of transmission, in-depth studies of *S. uberis* populations are essential to determine the best practices to control this pathogen. In this work, we optimized and validated a dot blot platform, combined with automatic image analysis, to rapidly assess the population structure of infective *S. uberis*, and evaluated its efficiency when compared to multilocus sequence analysis (MLSA) genotyping. Two dairy herds with prevalent *S. uberis* infections were followed in a 6 month period, in order to collect and characterize isolates from cows with persistent infections. These herds, located in Portugal (Barcelos and Maia regions), had similar management practices, with the herd from Barcelos being smaller and having a better milking parlor management, since infected cow segregation was immediate. A total of 54 *S. uberis* isolates were obtained from 24 different cows from the two herds. To overcome operator-dependent analysis of the dot blots and increase the technique's consistency and reliability, the hybridization signals were converted into probability values, with average probabilities higher than 0.5 being considered positive results. These data allowed to confirm the isolates' identity as *S. uberis* using taxa-specific markers and to determine the presence of virulence- and antibiotic resistance-related genes. In addition, MLSA allowed to disclose the most prevalent *S. uberis* clonal lineages in both herds. Seven different clusters were identified, with Barcelos showing a high clonal diversity and Maia a dominant lineage infecting most cows, suggesting distinct epidemiological patterns, with *S. uberis* displaying an environmental or contagious transmission pattern depending on the herd. Overall, this work showed the utility of dot blot and MLSA to characterize population structure and epidemiological patterns of mastitis-causing *S. uberis*. This approach allowed to disclose prevalent virulence patterns and clonal lineages of *S. uberis* in two distinct herds, and gain insights on the impact of herd management practices on pathogen population structure.

## Introduction

Bovine mastitis is a disease characterized by mammary gland inflammation that affects dairy herds worldwide. Mastitis leads to a decline in milk production and quality, which coupled with high treatment costs or early culling of animals, is responsible for significant losses in dairy farms (Petrovski et al., 2006). Bovine mastitis can be classified as subclinical mastitis (asymptomatic) or clinical mastitis (symptomatic). Even though the inflammatory symptoms are evident in the case of clinical mastitis, prompting producers, or veterinarians to take appropriate action, diagnosis of subclinical mastitis is mainly carried out by milk testing, e.g., using the California Mastitis Test (Schalm and Noorlander, 1957). This can be problematic for farmers, since subclinical infections, which can go unnoticed without regular cow screenings, are associated with a higher somatic cell count in milk. This high cell count results in decreased milk quality and economic value (Halasa et al., 2007), which implies that disease control and prevention are essential for dairy farmers' subsistence. Mastitis control programs are fundamentally based on three pillars: (a) prevention of new infections, (b) elimination of existing ones, and (c) monitoring udder health, with disease prevention taking a predominant role in recent years (LeBlanc et al., 2006). Both the “five point plan” aimed at lowering the overall incidence of mastitis (McDougall, 2002), and the use of vaccination (Finch et al., 1997) can be considered advantageous to minimize losses attributed to this disease.

Several bacterial species can cause the disease and typical bovine mastitis-causing pathogens include *Staphylococcus aureus, Escherichia coli*, and members of the *Streptococcus* genus (*Streptococcus uberis, Streptococcus agalactiae*, and *Streptococcus dysgalactiae*) (Bradley, 2002). Among these, *Streptococcus uberis* (*S. uberis*) is acknowledged as one of the most important mastitis pathogens, since it is frequently isolated from dairy herds worldwide (Riekerink et al., 2008; Verbeke et al., 2014). Although largely regarded as an environmental pathogen, characterized by different clonal lineages causing disease (Lundberg et al., 2014), *S. uberis* can also behave as a contagious pathogen (Zadoks et al., 2001; Rato et al., 2008). The typical environmental reservoirs where *S. uberis* can be found include grass, straw bedding and also the skin, gut, and genital tract of cattle (Leigh, 1999; Lopez-Benavides et al., 2007). The ability of this pathogen to survive in a wide range of substrates decreases the effectiveness of typical herd hygiene and disease prevention methods, since these are mostly aimed at controlling contagious pathogens. These drawbacks call for the need to develop and optimize new procedures to support disease prevention caused by both contagious and environmental pathogens, given that the factors influencing their prevalence are not identical (Barkema et al., 1999).

Despite the fact that antibiotic treatment is still extensively employed in the control of bovine mastitis, the use of the correct antibiotic and duration of treatment are essential to ensure a successful therapy (Hillerton and Kliem, 2002; Swinkels et al., 2015). Furthermore, antibiotic resistance in pathogens remains a great concern, making regular screenings of antibiotic resistance patterns an indispensable procedure (Erskine et al., 2002). Indeed, resistance of *S. uberis* to conventional antibiotics has been previously reported (León-Galván et al., 2015), adding to the difficulty to control this pathogen in herds.

The ability of *S. uberis* strains to effectively cause disease has been linked to the presence of specific virulence traits, which might provide an advantage in outcompeting other bacteria, or an improved capacity to invade and survive in the teat environment. In recent years, many of the genes coding for these traits were identified, namely the nisin U operon. This operon is responsible for the production and immunity to a bacteriocin of the lantibiotic class, which has antimicrobial activity against many lactic acid bacteria (Wirawan et al., 2006). Furthermore, the enhanced growth in the teat environment has been associated with the presence of a plasminogen activator protein, required for the degradation of extracellular matrix proteins (Rosey et al., 1999), and oligopeptide permeases that promote the ability to obtain essential amino acids from milk peptides (Smith et al., 2002). Efficient colonization and survival can also be attributed to the protein SUAM (*Streptococcus uberis* adhesion molecule), which plays a role in adherence to the bovine mammary epithelial cells (Almeida et al., 2006); to the ability of this species to produce an hyaluronic acid capsule, which confers resistance to phagocytosis and desiccation in the environment (Ward et al., 2001; Field et al., 2003); and to a glyceraldehyde-3-phosphate dehydrogenase (GAPDH) able to bind host proteins and protect against reactive oxygen species (Reinoso et al., 2011). The first fully sequenced genome of *S. uberis* (strain 0140J) confirmed the presence of several genes consistent with an organism capable of surviving in several environmental niches (Ward et al., 2009) and opened the possibility of using comparative genomic analyses to identify further virulence factors (Hossain et al., 2015).

Epidemiology studies are essential to study *S. uberis* populations and to identify problematic clonal lineages and preferred modes of transmission, which may vary in different dairy herds (Zadoks et al., 2003; Davies et al., 2016). Although fingerprinting techniques, such as Pulsed-field gel electrophoresis (Lundberg et al., 2014), can be used for this purpose, sequence-based typing methods such as multilocus sequence analysis (MLSA) and multilocus sequence typing (MLST) present several advantages, especially concerning inter-laboratory comparison of data. Two MLST schemes were developed for *S. uberis*, one that includes both housekeeping genes and virulence genes (Zadoks et al., 2005), and one consisting of seven housekeeping genes (Coffey et al., 2006).

In this work, a dot blot platform coupled with automatic data analysis was used to study *S. uberis* populations obtained from selected cows with recurring mastitis from two herds in Northern Portugal (Barcelos and Maia regions), aiming to provide insights of prevalent virulence patterns and clonal lineages and gain further insights on the impact of herd management practices on *S. uberis* population clonal structure of each herd.

## Materials and methods

### Collection of *S. uberis* isolates from milk samples

*S. uberis* isolates were obtained from milk samples of infected cows from two intensive dairy farms located in Northern Portugal (Barcelos and Maia). The farms were followed from December 2013 to May 2014.

In the first visit, all lactating milking cows were clinically evaluated by a veterinarian, namely for the presence of swollen and red quarters, and the California Mastitis Test (CMT) was carried out at quarter level. Composite milk samples from the four quarters were collected in the first visit to identify *S. uberis* infected cows. The cows infected with *S. uberis* in the first visit were monitored in the following months for the presence of *S. uberis* by taking quarter level samples and recording CMT results and somatic cell counts (SCC). Each time a new cow entered the milking herd, after parturition, the above mentioned procedure was initiated.

For bacterial isolation, milk samples were collected as follows: (i) discarding first strips of milk; (ii) disinfection of the teat end by scrubbing with cotton patches soaked in 70% alcohol; (iii) collection of milk into a sterile plastic vial; (iv) proper identification of the vial to trace the correspondent quarter from each specific cow; (v) placing the vial in a cool container (<8°C) to be transported to the lab. Procedures recommended by the National Mastitis Council for milk collection were followed (Oliver et al., 2004). For quarter level sampling, 20 mL of milk were collected, while for composite samples, 5 mL of milk from each quarter were collected. Identification of bacterial isolates using the VITEK 2 system (bioMérieux, Durham, NC) and quantification of SCC in the collected milk samples were carried out at SEGALAB (Laboratório de Sanidade Animal e Segurança Alimentar, S.A).

A total of 54 *S. uberis* isolates were selected for this study (Table 1), with a subset of 44 isolates obtained from 14 animals at different dates.

**Table 1:** *****Streptococcus uberis*** isolates used in this work**.

**Strain**	**Animal number**	**Collection date**	**Herd**	**Quarter^a^**	**SCC (× 10^3^)^b^**	**Dot blot pattern**	**MLSA type**
SU57	1	06-01-2014	Barcelos	FR^*^	12,695	A	I
SU91	1	13-03-2014	Barcelos	FR	2417	A	
SU114	1	09-05-2014	Barcelos	All	135	A	I
SU16	2	18-01-2013	Barcelos	–	541	A	VII
SU112	2	09-05-2014	Barcelos	All	302	B	VI
SU52	3	13-12-2013	Barcelos	FR	307	C	IV
SU53	3	13-12-2013	Barcelos	FL	230	C	
SU58	3	06-01-2014	Barcelos	FR	374	C	
SU59	3	06-01-2014	Barcelos	FL	216	C	IV
SU90	4	13-03-2014	Barcelos	RR	5498	D	IIIa
SU113	4	09-05-2014	Barcelos	All	3457	D	IIIa
SU76	5	29-01-2014	Maia	FR	201	E	II
SU80	5	25-02-2014	Maia	FR	142	E	II
SU72	6	29-01-2014	Maia	RR	165	E	
SU73	6	29-01-2014	Maia	FL	1677	A	IIIb
SU89	6	25-02-2014	Maia	RL	1156	E	II
SU70	7	29-01-2014	Maia	FR	295	E	
SU86	7	25-02-2014	Maia	FR	193	E	
SU63	8	29-01-2014	Maia	FR	456	E	II
SU64	8	29-01-2014	Maia	RR	1309	E	
SU65	8	29-01-2014	Maia	FL	223	E	II
SU82	8	25-02-2014	Maia	FR	1742	E	
SU83	8	25-02-2014	Maia	RR	179	F	II
SU67	9	29-01-2014	Maia	FR	321	E	II
SU103	9	09-05-2014	Maia	All	334	E	II
SU40	10	26-11-2013	Maia	RR	1416	E	II
SU62	10	29-01-2014	Maia	All	747	E	
SU79	10	25-02-2014	Maia	RR	731	E	
SU99	10	09-05-2014	Maia	All	385	E	II
SU45	11	26-11-2013	Maia	RL	–	G	V
SU101	11	09-05-2014	Maia	All	1151	E	II
SU42	12	26-11-2013	Maia	FR	519	E	II
SU43	12	26-11-2013	Maia	FL	372	E	
SU69	12	29-01-2014	Maia	FR	3169	E	
SU85	12	25-02-2014	Maia	FR	2122	E	II
SU49	13	26-11-2013	Maia	RL	173	E	II
SU68	13	29-01-2014	Maia	RR	799	E	II
SU87	13	25-02-2014	Maia	RR	562	E	II
SU88	13	25-02-2014	Maia	RL	1748	E	
SU104	13	09-05-2014	Maia	All	1238	E	II
SU60	14	29-01-2014	Maia	RR^*^	540	E	II
SU61	14	29-01-2014	Maia	FL	14,223	E	II
SU84	14	25-02-2014	Maia	FL	409	E	
SU98	14	09-05-2014	Maia	All	1519	E	II
SU41	15	26-11-2013	Maia	RL	419		V
SU46	16	26-11-2013	Maia	RL^*^	1696		IIIb
SU50	17	26-11-2013	Maia	FR	103		II
SU93	18	09-05-2014	Maia	All	51		II
SU95	19	09-05-2014	Maia	All	100		II
SU96	20	09-05-2014	Maia	All^*^	1156		II
SU97	21	09-05-2014	Maia	All	284		II
SU107	22	09-05-2014	Maia	All	727		II
SU109	23	09-05-2014	Maia	All	2410		II
SU110	24	09-05-2014	Maia	All	661		II

a Specific quarter from which the isolate was obtained: front right/rear right/front left/rear left (FR/RR/FL/RL). All, composite milk sample;

* *visible mastitis symptoms*.

b *SCC, somatic cell count (cells/mL)*.

### DNA extraction and PCR conditions

*S. uberis* isolates were cultured in Brain Heart Infusion (BHI; Becton, Dickinson and Company, Le Pont de Claix, France) at 37°C. DNA extraction from pure cultures was carried out using the E.Z.N.A Bacteria DNA kit (Omega Bio-Tek, Norcross, Georgia, USA), following the manufacturer's instructions. DNA samples were quantified using the Qubit 2.0 fluorometer (Invitrogen, Eugene, Oregon, USA).

Primers pairs for amplification of genes *sua* (DNA probe V4), *pauA* (V6), *ermB* (R1), *linB* (R2), and *tetS* (R3) were designed using Geneious® 7.1.7 (Biomatters, available from http://www.geneious.com/) and synthesized by STAB VIDA (Lisbon, Portugal; Table S1).

PCR reactions were carried out using DNA from *S. uberis* LMG 9465 (Almeida et al., 2013) for amplification of *pauA* (V6) and *ermB* (R1); *S. uberis* SU3 (Almeida et al., 2013) for *sua* (V4); and *S. uberis* SU63 for *hasA* (V7), *linB* (R2), and *tetS* (R3) (Table 2). The PCR mastermix contained 1 × DreamTaq Buffer (Thermo Scientific, Lithuania), 0.2 mM of each dNTP (Fermentas, Ontario, Canada), 0.2 μM of each primer, 1 U of DreamTaq DNA polymerase (Thermo Scientific) and 25 ng of bacterial DNA. The PCR conditions were as follows: initial denaturation of 5 min at 95°C, 35 cycles at 95°C for 30 s, 55°C for 30 s, and 72°C for 45 s and a final extension step of 10 min at 72°C. PCR products were visualized in 1% agarose gels and purified using the GFX PCR DNA and gel band purification kit (GE Healthcare, Buckinghamshire, United Kingdom). Identity of PCR products was confirmed by sequencing (STAB VIDA).

**Table 2:** **DNA probes used in this work**.

**Type**	**DNA marker**	**Description**	**References**
Taxonomic	U1	Taxa-specific (*S. uberis*)	Almeida et al., 2013
	U2	Taxa-specific (*S. uberis*)	Almeida et al., 2013
	A1	Taxa-specific (*S. agalactiae*)	Almeida et al., 2013
	A2	Taxa-specific (*S. agalactiae*)	Almeida et al., 2013
Nisin	NU1	Regulation gene (*nsuR*)	Almeida et al., 2013
operon	NU3	Nisin immunity gene (*nsuI*)	Almeida et al., 2013
Virulence related	V1	Hyaluronic acid operon gene (*hasC*)	Ward et al., 2001
	V2	Glyceraldehyde 3-phosphate dehydrogenase gene (*gapC*)	Reinoso et al., 2011
	V3	Oligopeptide permease gene (*oppF*)	Smith et al., 2002
	V4	*S. uberis* adhesion molecule gene (*sua*)	This work
	V6	Plasminogen activator gene (*pauA*)	This work
	V7	Hyaluronic acid operon gene (*hasA*)	Field et al., 2003
Antibiotic resistance	R1	Erythromycin resistance gene (*ermB*)	This work
	R2	Pirlimycin resistance gene (*linB*)	This work
	R3	Tetracycline resistance gene (*tetS*)	This work

### Characterization of isolates using multi locus sequence analysis (MLSA)

For genotyping, the sequence variation of genes *arc, ddl, gki, recP, tdk, tpi*, and *yqiL* (Coffey et al., 2006) was assessed in 987 *S. uberis* isolates (http://PubMLST.org/ suberis/) using Geneious® 7.1.7. The sequences were aligned using ClustalW and the number of unique sequences for each gene was determined. Genes *ddl, gki*, and *tdk* were selected as the most informative for MLSA genotyping.

PCR amplification of *ddl, gki*, and *tdk* was performed as mentioned above with DNA from 40 *S. uberis* isolates. The PCR products were sequenced (STAB VIDA) and the obtained sequences were concatenated using Geneious® 7.1.7. A total of 1027 concatenated sequences, comprising those obtained in this work and the ones available in the PubMLST database, were aligned using ClustalW, and a Maximum Likelihood tree was constructed in MEGA 6 (Tamura et al., 2013) using the Hasegawa-Kishino-Yano [HKY+G (0.33) + I (0.84)] with 2000 bootstrap replicates.

### Dot blot analysis

For Dot blot assays, 100 ng of the PCR products corresponding to markers V4 (*sua*), V6 (*pauA*) V7 (*hasA*), R1 (*ermB*), R2 (*linB*), and R3 (*tetS*) (Table 2) were labeled with digoxigenin using the DIG-High Prime DNA labeling kit (Roche), following the manufacturer's instructions. Final probe concentration was adjusted to 100 ng mL^−1^. Probes U1, U2, A1, A2, NU1 (*nsuR*), NU3 (*nsuI*), V1 (*hasC*), V2 (*gapC*), and V3 (*oppF*) were validated in a previous work (Almeida et al., 2013).

Hundred nanogram of DNA from each of the analyzed *S. uberis* (Table 1) were spotted on a nylon membrane using a bio dot apparatus (Bio Rad, Hercules, USA). Hybridization with the labeled probes was carried out overnight at 68°C with washing and detection steps carried out according to the DIG system recommendations (Roche). A Molecular Imager Chemi Doc system (Bio Rad) was used to acquire the dot blot images, which were quantified using a custom-made image analysis software (Caridade et al., 2015). The results obtained with this software, which outputs the probability of each dot being a positive hybridization signal, were used to calculate the average probability values obtained for each probe/strain DNA combination (Table 3). Probability values higher than 0.5 were considered as positive results.

**Table 3:** Average probability values of each dot blot hybridization result being a positive signal^*^.

	**DNA marker**															
	**Taxonomic**				**Nisin operon**		**Virulence related**						**Antibiotic resistance**			**Dot blot pattern**
**Strain**	**U1**	**U2**	**A1**	**A2**	**NU1**	**NU3**	**V1**	**V2**	**V3**	**V4**	**V6**	**V7**	**R1**	**R2**	**R3**	
					**(*nsuR*)**	**(*nsuI*)**	**(*hasC*)**	**(*gapC*)**	**(*oppF*)**	**(*sua*)**	**(*pauA*)**	**(*hasA*)**	**(*ermB*)**	**(*linB*)**	**(*tetS*)**	
SU57	**1.00**	**1.00**	0.00	0.00	0.02	0.08	**0.95**	**1.00**	**1.00**	**1.00**	**1.00**	**1.00**	0.05	0.01	0.01	**A**
SU91	**0.99**	**1.00**	0.00	0.00	0.21	0.05	**0.99**	**0.99**	**0.98**	**0.98**	**1.00**	**1.00**	0.05	0.04	0.02	**A**
SU114	**0.95**	**1.00**	0.00	0.00	0.00	0.05	**0.89**	**0.99**	**0.98**	**0.96**	**1.00**	**1.00**	0.09	0.02	0.02	**A**
SU16	**1.00**	**1.00**	0.00	0.00	0.03	0.14	**0.92**	**1.00**	**1.00**	**1.00**	**0.96**	**1.00**	0.04	0.03	0.06	**A**
SU112	**0.96**	**1.00**	0.00	0.00	0.11	0.04	**0.95**	**0.98**	**0.96**	**0.98**	**1.00**	0.00	0.17	**1.00**	0.10	**B**
SU52	**0.98**	**1.00**	0.01	0.00	**0.92**	**1.00**	**0.85**	**1.00**	**0.97**	**0.95**	**0.98**	0.00	**1.00**	0.01	0.00	**C**
SU53	**0.99**	**1.00**	0.00	0.00	**1.00**	**1.00**	**0.92**	**1.00**	**1.00**	**1.00**	**1.00**	0.00	**1.00**	0.02	0.01	**C**
SU58	**0.93**	**1.00**	0.00	0.00	**0.94**	**0.98**	**0.98**	**1.00**	**1.00**	**0.99**	**0.99**	0.00	**1.00**	0.01	0.06	**C**
SU59	**0.95**	**1.00**	0.00	0.00	**1.00**	**0.96**	**0.98**	**1.00**	**0.99**	**1.00**	**1.00**	0.00	**1.00**	0.02	0.10	**C**
SU90	**0.99**	**1.00**	0.03	0.05	0.00	0.04	**0.98**	**1.00**	**1.00**	**0.98**	**1.00**	**1.00**	0.00	0.03	**1.00**	**D**
SU113	**1.00**	**1.00**	0.00	0.02	0.07	0.05	**0.97**	**1.00**	**1.00**	**1.00**	**1.00**	**1.00**	0.03	0.01	**1.00**	**D**
SU76	**0.96**	**1.00**	0.00	0.00	0.01	0.05	**1.00**	**1.00**	**1.00**	**1.00**	**1.00**	**1.00**	0.05	**0.98**	**0.97**	**E**
SU80	**0.86**	**1.00**	0.00	0.00	0.07	0.02	**1.00**	**0.93**	**1.00**	**1.00**	**1.00**	**1.00**	0.00	**0.99**	**1.00**	**E**
SU72	**0.97**	**1.00**	0.00	0.00	0.03	0.05	**0.98**	**1.00**	**1.00**	**0.82**	**1.00**	**1.00**	0.03	**1.00**	**0.97**	**E**
SU73	**0.96**	**1.00**	0.01	0.00	0.01	0.05	**1.00**	**1.00**	**1.00**	**0.96**	**1.00**	**1.00**	0.01	0.01	0.00	**A**
SU89	**0.95**	**1.00**	0.00	0.00	0.15	0.05	**0.99**	**1.00**	**1.00**	**1.00**	**1.00**	**1.00**	0.04	**1.00**	**1.00**	**E**
SU70	**0.91**	**1.00**	0.00	0.01	0.02	0.03	**0.87**	**1.00**	**1.00**	**0.97**	**1.00**	**0.98**	0.21	**0.99**	**0.98**	**E**
SU86	**1.00**	**1.00**	0.00	0.13	0.11	0.12	**0.97**	**1.00**	**1.00**	**1.00**	**1.00**	**1.00**	0.13	**1.00**	**1.00**	**E**
SU63	**0.79**	**1.00**	0.01	0.04	0.01	0.04	**0.95**	**1.00**	**1.00**	**1.00**	**1.00**	**1.00**	0.13	**1.00**	**1.00**	**E**
SU64	**0.89**	**1.00**	0.02	0.05	0.02	0.03	**0.99**	**1.00**	**1.00**	**1.00**	**1.00**	**1.00**	0.05	**1.00**	**1.00**	**E**
SU65	0.20	**0.85**	0.02	0.06	0.00	0.01	**0.98**	**0.85**	**0.73**	**1.00**	**1.00**	**0.88**	0.03	**0.77**	**0.96**	**E**
SU82	**0.97**	**1.00**	0.01	0.08	0.21	0.06	**1.00**	**1.00**	**1.00**	**0.98**	**1.00**	**1.00**	0.05	**1.00**	**1.00**	**E**
SU83	**0.98**	**1.00**	0.01	0.03	0.15	0.06	**0.98**	**1.00**	**1.00**	**1.00**	**1.00**	**1.00**	0.05	**1.00**	0.24	**F**
SU67	**0.97**	**1.00**	0.00	0.02	0.02	0.04	**0.48**	**1.00**	**1.00**	**0.96**	**1.00**	**1.00**	0.09	**1.00**	**1.00**	**E**
SU103	**0.94**	**1.00**	0.00	0.00	0.09	0.04	**0.95**	**1.00**	**1.00**	**1.00**	**1.00**	**1.00**	0.00	**1.00**	**1.00**	**E**
SU40	**0.92**	**1.00**	0.00	0.02	0.01	0.03	**0.95**	**1.00**	**0.97**	**1.00**	**1.00**	**1.00**	0.00	**1.00**	**1.00**	**E**
SU62	**0.92**	**1.00**	0.01	0.05	0.01	0.06	**1.00**	**1.00**	**1.00**	**1.00**	**1.00**	**1.00**	0.03	**1.00**	**1.00**	**E**
SU79	**0.64**	**1.00**	0.00	0.00	0.18	0.02	**0.85**	**0.97**	**0.97**	**1.00**	**1.00**	**1.00**	0.04	**1.00**	**1.00**	**E**
SU99	**0.95**	**0.99**	0.00	0.00	0.18	0.07	**0.99**	**0.94**	**0.98**	**1.00**	**1.00**	**1.00**	0.05	**1.00**	**1.00**	**E**
SU45	**1.00**	**1.00**	0.00	0.04	**0.94**	**1.00**	**0.99**	**1.00**	**1.00**	**1.00**	**1.00**	**1.00**	**1.00**	0.07	0.00	**G**
SU101	**0.88**	**1.00**	0.00	0.02	0.04	0.04	**0.95**	**1.00**	**1.00**	**0.99**	**1.00**	**1.00**	0.07	**1.00**	**1.00**	**E**
SU42	**0.92**	**1.00**	0.00	0.01	0.00	0.06	**0.95**	**1.00**	**1.00**	**1.00**	**0.97**	**1.00**	0.03	**1.00**	**1.00**	**E**
SU43	**0.98**	**1.00**	0.01	0.00	0.01	0.07	**0.98**	**1.00**	**1.00**	**1.00**	**1.00**	**1.00**	0.01	**1.00**	**1.00**	**E**
SU69	**0.89**	**1.00**	0.02	0.07	0.01	0.04	**1.00**	**1.00**	**1.00**	**0.94**	**1.00**	**1.00**	0.00	**1.00**	**1.00**	**E**
SU85	**0.98**	**1.00**	0.00	0.00	0.05	0.07	**0.99**	**1.00**	**0.94**	**1.00**	**1.00**	**1.00**	0.00	**0.95**	**1.00**	**E**
SU49	**0.94**	**1.00**	0.00	0.00	0.02	0.03	**0.94**	**1.00**	**1.00**	**1.00**	**1.00**	**1.00**	0.00	**0.99**	**0.76**	**E**
SU68	**0.91**	**1.00**	0.00	0.00	0.03	0.03	**0.92**	**1.00**	**0.99**	**0.91**	**1.00**	**1.00**	0.00	**0.96**	**1.00**	**E**
SU87	**0.96**	**1.00**	0.00	0.00	0.13	0.04	**0.99**	**1.00**	**1.00**	**1.00**	**1.00**	**1.00**	0.05	**1.00**	**1.00**	**E**
SU88	**0.82**	**0.99**	0.00	0.00	0.02	0.03	**1.00**	**0.98**	**0.94**	**1.00**	**1.00**	**1.00**	0.00	**1.00**	**1.00**	**E**
SU104	**0.85**	**0.93**	0.01	0.00	0.05	0.05	**0.96**	**1.00**	**1.00**	**1.00**	**1.00**	**1.00**	0.03	**0.99**	**1.00**	**E**
SU60	**0.78**	**0.99**	0.00	0.00	0.03	0.02	**1.00**	**0.99**	**0.97**	**1.00**	**1.00**	**1.00**	0.03	**0.97**	**0.78**	**E**
SU61	**0.91**	**1.00**	0.00	0.00	0.01	0.03	**0.98**	**0.99**	**1.00**	**1.00**	**1.00**	**1.00**	0.01	**0.99**	**0.98**	**E**
SU84	**1.00**	**1.00**	0.05	0.02	0.11	0.07	**0.98**	**1.00**	**1.00**	**1.00**	**1.00**	**1.00**	0.00	**1.00**	**1.00**	**E**
SU98	**0.92**	**1.00**	0.00	0.02	0.09	0.03	**1.00**	**1.00**	**1.00**	**1.00**	**1.00**	**1.00**	0.00	**0.92**	**1.00**	**E**

* *Probability values higher than 0.5 (considered as positive results) are highlighted in bold*.

### Epidemiological criteria

Cows were considered subclinically infected if *S. uberis* were isolated from milk samples, and clinically infected when symptoms of inflammation and/or abnormal secretions were identified. Infected cows were considered persistently infected when *S. uberis* with the same MLSA type were isolated: (i) two or more times in the same quarter, (ii) in a composite sample and in a quarter sample, and (iii) from a cow which dried off and calved. New cases of mastitis were assigned to cows which entered the milking herd and were found infected by *S. uberis* regardless the MLSA type.

## Results

### Characterization of milk samples and isolation of *S. uberis*

Visits to herds in the Barcelos and Maia regions, between December 2013 and May 2014, allowed to obtain 54 *S. uberis* isolates from 24 different cows (cow #1 to cow #24, Table 1). All isolates were obtained from cows with sub-clinical infection except for four cows (#1, #14, #16, and #20), which showed clinical infection signs. Among the total of 24 infected cows analyzed in this work, dry quarters were observable in four animals (#3, #10, #16, and #23).

Somatic cell counts varied considerably between individuals, with the highest values registered for cows #1 and #14 (12,695 × 10^3^ and 14,223 × 10^3^ cells mL^−1^, respectively, indicative of heavy inflammation). SCC in 45 samples (83%) was higher than 200 × 10^3^ cells mL^−1^, a standardized threshold indicative of infected quarters (Schukken et al., 2003), meaning that 17% of the infected milk samples would not have been detected if the criteria for sampling was SCC > 200 × 10^3^ cells mL^−1^.

Ten *S. uberis* isolates from the Maia herd were obtained from 10 animals (#15–#24) which entered in lactation during the 6 month period and were considered new infections (Table 1).

### Genotyping of *S. uberis* isolates

The preliminary assessment of the seven MLST genes proposed by Coffey et al. (2006) showed that genes *ddl, gki*, and *tdk* are the most informative for genotyping, with 0.104, 0.095, and 0.098 variations per nucleotide and 48, 44, and 72 unique sequences, respectively.

**Figure 1:**
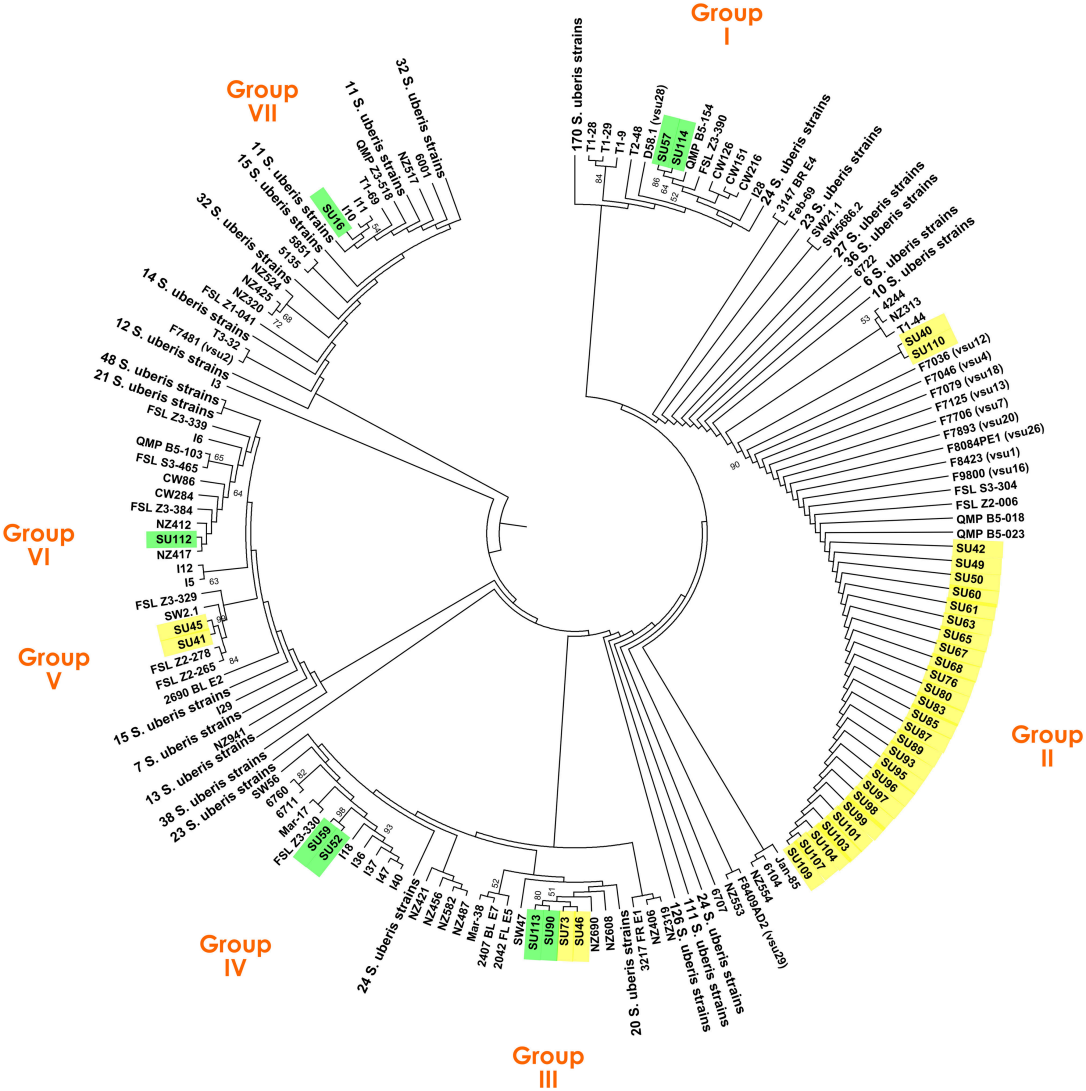
**Maximum Likelihood tree based on the concatenated sequences of genes ***ddl***, ***gki***, and ***tdk*** of a total of 1027 ***S. uberis*** strains, including 40 isolates obtained in this study**. *S. uberis* isolates from the Barcelos herd are highlighted in green, and from the Maia herd in yellow.

The clonal diversity of a subset of 40 *S. uberis*, representative of the isolate diversity obtained in this work (Table 1), was inferred through a comprehensive ML analysis using the concatenated sequences of genes *ddl, gki*, and *tdk* retrieved from the PubMLST database from a total of 987 strains. This analysis resulted in splitting *S. uberis* isolates in seven clusters (Figure 1), six of which contained four or less isolates, with the following distribution: Group I—isolates SU57 and SU114 (obtained from cow #1, Barcelos); Group III—isolates SU113 and SU90 (cow #4, Barcelos) and SU73 and SU46 (cows #6 and #16, respectively, Maia); Group IV—isolates SU59 and SU52 (cow #3, Barcelos); Group-V—isolates SU45 and SU41 (cow #11 and cow #15, respectively, Maia); Groups VI and VII- isolates SU112 and SU16 (cow #2, Barcelos). The dominant cluster (Group II) contained 28 *S. uberis* isolated from 16 different cows in Maia.

To determine if the same gene alleles have been previously identified, the sequences obtained for all the isolates in this work were compared with the Sequence Types (STs) available at the PubMLST database (Table S2). This analysis revealed that most of the gene sequences had corresponding ST alleles in the database. The exceptions were the new sequences obtained for *ddl* in isolates SU113, SU90, SU73, and SU46; for *gki* in isolate SU40 and for *tdk* in isolates SU113, SU90, SU52, SU59, SU45, and SU41. Interestingly, isolates clustered in Groups II had identical alleles to Portuguese strains isolated in 2002. All nucleotide sequences obtained in this work were submitted to the NCBI database with accession numbers KU758715 to KU758834 and to PubMLST (*ddl* allele 56; *gki* allele 55; *tdk* alleles 89, 90, and 91).

### Dot blot analysis

**Figure 2:**
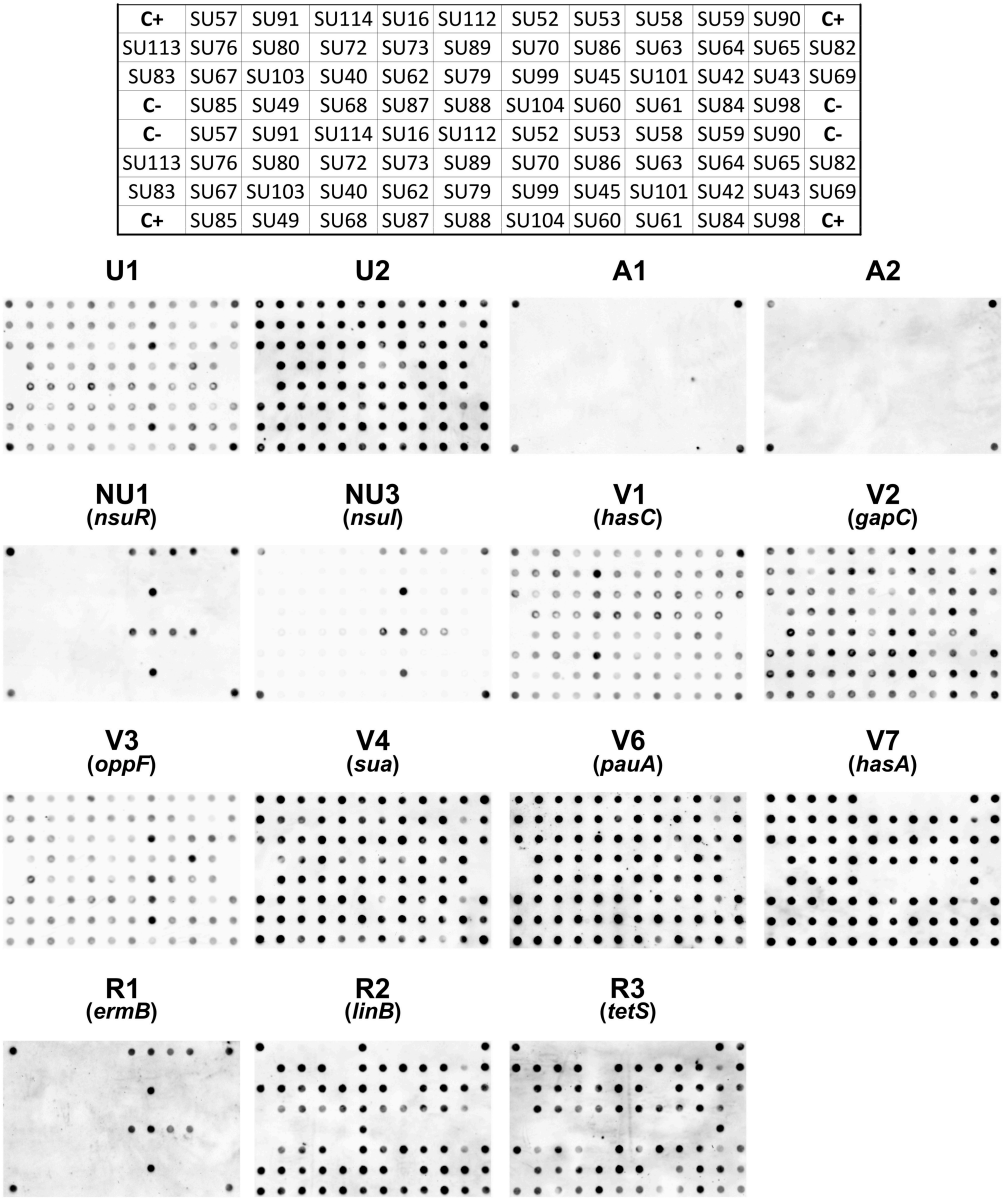
**Dot blots using 15 probes and genomic DNA from 44 ***S. uberis*** isolates**. The following groups of probes were used: Taxonomic probes (U1, U2, A1, A2); nisin operon (NU1, NU3); virulence factors (V1, V2, V3, V4, V6, V7) and antibiotic resistance (R1, R2, R3). The top grid represents the position of the DNA from each *S. uberis* isolate in the nylon membrane. C+ DNA used as template for probe labeling; C− TE buffer.

A total of 15 DNA markers were analyzed in this work: four taxonomic markers (U1 and U2, specific to *S. uberis*, and A1 and A2, specific for *S. agalactiae*) for confirmation of VITEK 2 identification of the isolates as *S. uberis*; two markers targeting the nisin operon (NU1-*nsuR* and NU3-*nsuI*); six markers designed for genes associated to increased virulence of *S. uberis* (V1-*hasC*, V2-*gapC*, V3-*oppF*, V4-*sua*, V6-*pauA*, V7-*hasA*) and three markers to assess resistance to antibiotics (R1-*ermB*, R2-*linB* and R3-*tetS*) (Table 2). The presence of these 15 genes in 44 *S. uberis* representative of the different MLSA types and obtained from the same animal at different dates was assessed using a dot blot platform (Figure 2).

Using a dedicated image analysis software previously described (Caridade et al., 2015), the dot blot results were converted in probability values of each dot being a positive result (Table 3). To ensure high confidence, only probability values higher than 0.5 were considered positive. Using this threshold, the results confirmed the identity of all isolates as *S. uberis*, with positive hybridization results being obtained with *S. uberis* specific markers U1 and U2 and no hybridization was observed with *S. agalactiae* specific markers A1 and A2.

The presence of the nisin U operon was addressed using probes NU1 and NU3, which targeted the regulation gene (*nsuR*) and the nisin immunity gene (*nsuI*), respectively. Results showed that this operon was present in a restricted number of isolates in the analyzed *S. uberis* population, as only five isolates contained the two genes (SU52, SU53, SU58, SU59 isolated in Barcelos and SU45 in Maia).

Five out of the six virulence-related probes tested (V1, V2, V3, V4, and V6) provided consistent positive results across all 44 tested isolates, showing that the genes *hasC, gapC, oppF, sua*, and *pauA*, respectively targeted by these markers, were present in all isolates. The exception was the *hasA* gene (DNA marker V7), which was absent from five isolates (SU112, SU52, SU53, SU58, and SU59 isolated in Barcelos).

The markers used to assess antibiotic resistance showed a greater variation amongst isolates. Probe R1 provided positive results with only five isolates (SU52, SU53, SU58, SU59, and SU45), suggesting a restricted distribution of the *ermB* gene (erythromycin resistance), identical to the one obtained for the nisin operon. Probe R2 (*linB* gene, pirlimycin resistance) and probe R3 (*tetS* gene, tetracycline resistance) both hybridized with a total of 32 isolates, with isolates SU112 and SU83 only providing positive results with the R2 probe, and isolates SU90 and SU113 with the R3 probe. The three antibiotic resistance-related genes tested were not detected in five isolates: SU57, SU91, SU114, and SU16 isolated from Barcelos, and SU73 isolated from Maia.

### Comparison between *S. uberis* MLSA types and their dot blot profiles

Dot blot results were compared with MLSA to assess the discriminatory ability of the obtained hybridization patterns (Figures 1, 2, Table 1). Dot blot patterns D, C, G, and B showed complete agreement with MLSA types IIIa, IV, V, and VI, respectively. Patterns E and F were exclusive to MLSA type II; however, it should be mentioned that dot blot pattern F is similar to pattern E, but with a probability value below 0.5 for the R3 probe (Table 3). Only dot blot pattern A presented different MLSA correspondences (types I, IIIb, and VII).

## Discussion

The difficult control of *S. uberis* makes these bacteria one of the most damaging pathogens in dairy herds, and emphasizes the importance of epidemiological studies to identify the most problematic clonal lineages, modes of transmission, and population structure in a given region.

In this work, two dairy herds located in Northern Portugal (Barcelos and Maia) were selected due to previous reported persistent *S. uberis* infections. The two herds had similar management practices, with the herd from Barcelos having roughly half the number of cows and a better milking parlor management, since infected cows were immediately segregated. In Barcelos, it was possible to obtain 11 isolates from four different cows. Concerning SCC, cows #1 and #4 had higher values, nevertheless SCC values of cow #1 were continuously decreasing throughout the sampling timeframe, with the last obtained value below 200.000 cells mL^−1^. A total of 43 isolates were obtained from Maia, 33 of which representing persistent infections, from 20 cows. SCC varied considerably between individuals and within the same animal during the sampling timeframe (Table 1).

The MLSA genotyping (Figure 1) showed that while in Maia most cows were infected by lineages within the same clade (Group II), in Barcelos each cow was generally infected by different lineages, clustered in five groups (Groups I, IIIa, IV, VI, and VII). These results suggest that in Barcelos *S. uberis* has the typical pattern of an environmental pathogen, since transmission between cows was not observed, and with different genotypes isolated from individual cows (Lundberg et al., 2014). On the other hand, it appears that in Maia *S. uberis* behaves as a contagious and persistent pathogen, suggesting cow-to-cow transmission, even though this could also be explained by the continuous acquisition from the same environmental source (Rato et al., 2008). Persistent infections of cows #5, #8, #9, #10, #12, #13, and #14 with isolates from Group II, suggests the ability of this lineage of *S. uberis* to colonize the quarter for long periods. Additionally, many of the new infections, namely in cows #6, #8, and #13, were caused by MLSA type II. The hypothesis of this lineage being highly contagious is supported by the fact that cows #18–#24, with no recorded mastitis in the previous visits, were newly infected by Group II isolates within the 6 month sampling timeframe. These results further support that the different modes of transmission displayed by *S. uberis*, either environmental or contagious, are strongly dependent on the dairy herd analyzed (Zadoks et al., 2001).

The assessment of MLSA genotyping results allowed to compare the obtained gene sequences with those already publicly available (Table S2). This comparison showed that the majority of the gene sequences used for MLSA had already been identified in previous studies and available in the PubMLST database. Namely, the set of sequences corresponding to the Group II isolates, widely established in Maia, were already reported in Portugal in 2002, suggesting that this lineage is well-established in the country. Concerning *S. uberis* lineage persistence across time, in Maia it was noticeable that even though most recurring infections seem to be caused by the same clonal lineage, different *S. uberis* clones can also be responsible for persistent infections in the same cow, with only 1 month apart between isolation dates (cow #6 and #11). This indicates that these cows were initially infected with an environmental *S. uberis* and afterwards were infected by a contagious *S. uberis* belonging to Group II, prevalent in the herd. From this evidence one can assume that both environmental and contagious mastitis affect the Maia herd.

The analysis of the presence of relevant virulence-related features using dot blot is a valuable tool to understand which genes might play an important role in increased virulence and survival of *S. uberis*. In this work, 15 probes were used to rapidly screen the presence of relevant genes in the isolated *S. uberis* (Figure 2, Tables 2, 3). Four probes (U1, U2, A1, and A2) confirmed the isolate's identity as *S. uberis* by VITEK 2, a widely used phenotypic identification system that despite its usefulness shows some percentage of misidentifications (Di Domenico et al., 2015). The virulence-related genes *hasA* (probe V7), *hasC* (V1), *gapC* (V2), *oppF* (V3), *sua* (V4), and *pauA* (V6) were present in most of the *S. uberis* tested causing recurring infections, suggesting their importance for efficient *S. uberis* colonization. The majority of isolates contained the genes *hasC* (V1) and *hasA* (V7), associated with hyaluronic capsule production, corresponding to the typical pattern of genes *hasABC* found in fully sequenced strains (Hossain et al., 2015). Nevertheless, in five isolates from Barcelos the *hasA* (probe V7) gene was not detected. Since this gene is acknowledged as essential for capsule production, these results corroborate previous studies suggesting that acapsulate bacteria can cause infection (Field et al., 2003; Pullinger et al., 2006). The presence of *gapC* (V2) in all isolates reinforced the predictions of its importance in virulence (Reinoso et al., 2011). Genes *pauA* (V6) and *oppF* (V3) were also present in all isolates tested, emphasizing previous results showing their contribution to an enhanced growth in milk (Rosey et al., 1999; Smith et al., 2002). The importance of the protein SUAM, which mediates *S. uberis* adhesion (Almeida et al., 2006), was also corroborated, since the *sua* gene (V4) was detected in all isolates. Furthermore, the presence of the nisin operon (NU1-*nsuR*, NU3-*nsuI*), which can provide a competitive advantage (Pryor et al., 2009; Richards et al., 2011), was restricted to six out of 44 isolates in the analyzed population.

Dot blot results showed that the high SCC values recorded for some milk samples and SCC variation observed at different milk sampling times (Table 1) could not be ascribed to specific virulence patterns, suggesting that other factors play a decisive role in determining the severity of the inflammation.

Regarding antibiotic resistance, genes *linB* (pirlimycin resistance) and *tetS* (tetracycline resistance) were widespread in the Maia population, confirming previous studies showing their prevalence among *S. uberis* strains (Rato et al., 2013). However, in Barcelos, among the 11 obtained isolates, the presence of these genes was restricted to one isolate with *linB* (probe R2) and two with *tetS* (R3). Interestingly, the presence of gene *ermB* (probe R1) was highly infrequent and shared a matching pattern to the presence of *nsuR* (NU1) and *nsuI* (NU3), although the five isolates presenting the gene (SU52, SU53, SU58, SU59, and SU45) were not clustered in the same MLSA group (Figures 1, 2).

In total, seven different virulence gene patterns were found in this work (Table 3), confirming that isolates with different virulence patterns can be equally efficient in causing disease (Reinoso et al., 2011). Furthermore, dot blot results showed that isolates from each herd presented differentiating gene patterns, with patterns B, C, and D being unique to the Barcelos herd and patterns E, F, and G being unique to Maia. Gene pattern A, found in isolates SU57, SU91, SU114, SU16, and SU73, was the only pattern common to isolates from both herds.

In this study we characterized the epidemiology of *S. uberis* in two dairy herds using two different molecular approaches. The seven dot blot patterns obtained (A–G) provided a solid distinction of the different clonal lineages disclosed using MLSA (I–VII), indicating that the epidemiological patterns specific to each farm were discernible using the dot blot platform. Furthermore, dot blot results also allowed to discern important virulence and antibiotic resistance traits of the studied population, which is particularly useful for veterinarians. Overall, this data indicates that the chosen DNA markers and dot blot platform with automatic data analysis, were shown to be an efficient tool for a preliminary epidemiological assessment of *S. uberis*, which might be particularly useful for certified labs.

## Author contributions

PA, AA, IP, AP, MV, and FD performed the molecular biology experiments and *in silico* analyses. AP, MV, and HM performed the collection and analyses of milk samples and isolation of *S. uberis* strains. NR, HM, and FT were responsible for the conception and design of the work. All authors contributed to the drafting and revision of the manuscript.

### Conflict of interest statement

The authors declare that the research was conducted in the absence of any commercial or financial relationships that could be construed as a potential conflict of interest.
