# Supplementary material for: Application of a Dot Blot Hybridization Platform to Assess Streptococcus uberis Population Structure in Dairy Herds
Source: Front Microbiol. 2017 Jan 24;8:54. doi: 10.3389/fmicb.2017.00054 (PMC5258699; doi:10.3389/fmicb.2017.00054)
Supplement: Supplementary file 1 [file Table1.DOCX]

| **DNA**  **Marker** | **Target Gene** | **Genome** | **Forward primer (5´-3´)** | **Reverse Primer (5´-3´)** | **Amplicon Length (bp)** |
| --- | --- | --- | --- | --- | --- |
| U1 | SUB_RS08480 (SUB1704) | *S.* *uberis* 0140J | TCGTTTGTATACGCTTGATGCT | CACGTCTCTATAAAAGGAATTCCC | 229 |
| U2 | SUB_RS05985 (SUB1195) | *S.* *uberis* 0140J | TCCAGTTATGGTGACGCAATATGAT | GCTAAACTAGTATTCGGATGGGCTG | 333 |
| A1 | SAG2053 | *S.* *agalactiae* 2603V/R | ATGTAGCTGCTGATTCTGTCATAA | AATAGCTGGTGTAGATTTGACTGC | 314 |
| A2 | SAG2053 | *S.* *agalactiae* 2603V/R | ATGAACACAAAACAGCGTTTTTCA | AGTAGGTGTCTCATTTGCTATGCT | 192 |
| NU1 | *nsuR* | *S.* *uberis* strain 42 | CCAAGGTTGCAGCGCATTT | CCCCTTATTGTCTTGATGGGATT | 331 |
| NU3 | *nsuL* | *S.* *uberis* strain 42 | AATCAAATCGTTGATGAAAATGACC | AAACTTCTCCGTAATCCCAAACTTC | 502 |
| V1 | SUB_RS08420 (SUB1692) | *S. uberis* 0140J | TGCTTGGTGACGATTTGATG | GTCCAATGATAGCAAGGTACAC | 300 |
| V2 | SUB_RS08130 (SUB1630) | *S. uberis* 0140J | GCTCCTGGTGGAGATGATGT | GTCACCAGTGTAAGCGTGGA | 189 |
| V3 | SUB_RS08085 (SUB1621) | *S.* *uberis* 0140J | GGCCTAACCAAAACGAAACA | GGCTCTGGAATTGCTGAAAG | 419 |
| V4 | SUB_RS08150 (SUB1635) | *S.* *uberis* 0140J | TCAGTTGTTGTGATTGCTGACGTC | CAAACAAGTGGTTTCAGGTCCATT | 600 |
| V6 | SUB_RS08870 (SUB1785) | *S. uberis* 0140J | TTTTGGGAATATTTGGTTGTGC | TCAACCCGTTTTCTGAGAATAA | 427 |
| V7 | SUB_RS08445 (SUB1697) | *S.* *uberis* 0140J | GAAAGGTCTGATGCTGATG | TCATCCCCTATGCTTACAG | 319 |
| R1 | *ermB* | *S.* *uberis* strain 330 | AAAGCCATGCGTCTGACATC | TGTGGTATGGCGGGTAAGTT | 194 |
| R2 | *linB* | *S.* *uberis* iQMP Z3-369 | CCTGATACGAAGGCTATGCTT | GGTGACTTTGCAAATCCATAACT | 403 |
| R3 | *tetS* | *S.* *uberis* | AGGACAAACTTTCTGACGACA | CTGAATTGAGTTGTGTGGGTGA | 306 |

Table S1: Primer pairs used in this work
