# Supplementary material for: Application of a Dot Blot Hybridization Platform to Assess Streptococcus uberis Population Structure in Dairy Herds
Source: Front Microbiol. 2017 Jan 24;8:54. doi: 10.3389/fmicb.2017.00054 (PMC5258699; doi:10.3389/fmicb.2017.00054)
Supplement: Supplementary file 2 [file Table2.DOCX]

Table S2: Comparison of obtained sequences with the information available in the PubMLST database.

| **Group** | **Strain** | **Animal number** | **Collection date** | **Herd** | ***ddl*** | ***gki*** | ***tdk*** |
| --- | --- | --- | --- | --- | --- | --- | --- |
| I | SU57 | 1 | 06-01-2014 | Barcelos | 37 | 43 | 2 |
|  | SU114 | 1 | 09-05-2014 | Barcelos | 37 | 43 | 2 |
| II | SU76 | 5 | 29-01-2014 | Maia | 1 | 27 | 39 |
|  | SU80 | 5 | 25-02-2014 | Maia | 1 | 27 | 39 |
|  | SU89 | 6 | 25-02-2014 | Maia | 1 | 27 | 39 |
|  | SU63 | 8 | 29-01-2014 | Maia | 1 | 27 | 39 |
|  | SU65 | 8 | 29-01-2014 | Maia | 1 | 27 | 39 |
|  | SU83 | 8 | 25-02-2014 | Maia | 1 | 27 | 39 |
|  | SU67 | 9 | 29-01-2014 | Maia | 1 | 27 | 39 |
|  | SU103 | 9 | 09-05-2014 | Maia | 1 | 27 | 39 |
|  | SU40 | 10 | 26-11-2013 | Maia | 1 | 55 | 39 |
|  | SU99 | 10 | 09-05-2014 | Maia | 1 | 27 | 39 |
|  | SU101 | 11 | 09-05-2014 | Maia | 1 | 27 | 39 |
|  | SU42 | 12 | 26-11-2013 | Maia | 1 | 27 | 39 |
|  | SU85 | 12 | 25-02-2014 | Maia | 1 | 27 | 39 |
|  | SU49 | 13 | 26-11-2013 | Maia | 1 | 27 | 39 |
|  | SU68 | 13 | 29-01-2014 | Maia | 1 | 27 | 39 |
|  | SU87 | 13 | 25-02-2014 | Maia | 1 | 27 | 39 |
|  | SU104 | 13 | 09-05-2014 | Maia | 1 | 27 | 39 |
|  | SU60 | 14 | 29-01-2014 | Maia | 1 | 27 | 39 |
|  | SU61 | 14 | 29-01-2014 | Maia | 1 | 27 | 39 |
|  | SU98 | 14 | 09-05-2014 | Maia | 1 | 27 | 39 |
|  | SU50 | 17 | 26-11-2013 | Maia | 1 | 27 | 39 |
|  | SU93 | 18 | 09-05-2014 | Maia | 1 | 27 | 39 |
|  | SU95 | 19 | 09-05-2014 | Maia | 1 | 27 | 39 |
|  | SU96 | 20 | 09-05-2014 | Maia | 1 | 27 | 39 |
|  | SU97 | 21 | 09-05-2014 | Maia | 1 | 27 | 39 |
|  | SU107 | 22 | 09-05-2014 | Maia | 1 | 27 | 39 |
|  | SU109 | 23 | 09-05-2014 | Maia | 1 | 27 | 39 |
|  | SU110 | 24 | 09-05-2014 | Maia | 1 | 27 | 39 |
| IIIa | SU90 | 4 | 13-03-2014 | Barcelos | 56 | 5 | 89 |
|  | SU113 | 4 | 09-05-2014 | Barcelos | 56 | 5 | 89 |
| IIIb | SU73 | 6 | 29-01-2014 | Maia | 56 | 5 | 17 |
|  | SU46 | 16 | 26-11-2013 | Maia | 56 | 5 | 17 |
| IV | SU52 | 3 | 13-12-2013 | Barcelos | 2 | 43 | 90 |
|  | SU59 | 3 | 06-01-2014 | Barcelos | 2 | 43 | 90 |
| V | SU45 | 11 | 26-11-2013 | Maia | 16 | 5 | 91 |
|  | SU41 | 15 | 26-11-2013 | Maia | 16 | 5 | 91 |
| VI | SU112 | 2 | 09-05-2014 | Barcelos | 1 | 3 | 3 |
| VII | SU16 | 2 | 18-01-2013 | Barcelos | 2 | 4 | 28 |

Numbers represent the allele numbers from PubMLST matching the sequences obtained in this study for genes *ddl*, *tdk* and *gki*.
